# Unilateral hippocampal sparing during whole brain radiotherapy for multiple brain metastases: narrative and critical review

**DOI:** 10.3389/fonc.2024.1298605

**Published:** 2024-01-24

**Authors:** Petr Pospisil, Ludmila Hynkova, Lucie Hnidakova, Jana Maistryszinova, Pavel Slampa, Tomas Kazda

**Affiliations:** ^1^ Department of Radiation Oncology, Masaryk Memorial Cancer Institute, Brno, Czechia; ^2^ Department of Radiation Oncology, Faculty of Medicine, Masaryk University, Brno, Czechia

**Keywords:** whole brain radiotherapy, hippocampus, unilateral, brain metastases, neurocognitive function

## Abstract

**Background:**

The landscape of brain metastases radiotherapy is evolving, with a shift away from whole-brain radiotherapy (WBRT) toward targeted stereotactic approaches aimed at preserving neurocognitive functions and maintaining overall quality of life. For patients with multiple metastases, especially in cases where targeted radiotherapy is no longer feasible due to widespread dissemination, the concept of hippocampal sparing radiotherapy (HA_WBRT) gains prominence.

**Methods:**

In this narrative review we explore the role of the hippocampi in memory formation and the implications of their postradiotherapy lateral damage. We also consider the potential advantages of selectively sparing one hippocampus during whole-brain radiotherapy (WBRT). Additionally, by systematic evaluation of relevant papers published on PubMed database over last 20 years, we provide a comprehensive overview of the various changes that can occur in the left or right hippocampus as a consequence of radiotherapy.

**Results:**

While it is important to note that various neurocognitive functions are interconnected throughout the brain, we can discern certain specialized roles of the hippocampi. The left hippocampus appears to play a predominant role in verbal memory, whereas the right hippocampus is associated more with visuospatial memory. Additionally, the anterior part of the hippocampus is more involved in episodic memory and emotional processing, while the posterior part is primarily responsible for spatial memory and pattern separation. Notably, a substantial body of evidence demonstrates a significant correlation between post-radiotherapy changes in the left hippocampus and subsequent cognitive decline in patients.

**Conclusion:**

In the context of individualized palliative radiotherapy, sparing the unilateral (specifically, the left, which is dominant in most individuals) hippocampus could expand the repertoire of strategies available for adapted WBRT in cases involving multiple brain metastases where stereotactic radiotherapy is not a viable option. Prospective ongoing studies assessing various memory-sparing radiotherapy techniques will define new standard of radiotherapy care of patients with multiple brain metastases.

## 1 Introduction

Brain metastases (BM) are the most common intracranial tumors in adults, accounting for more than half of all brain tumors. The incidence of BM is steadily increasing, primarily due to advances in comprehensive cancer care, better control of extracranial disease through improved systemic therapy, and enhanced detection of small metastases using more easily accessible magnetic resonance imaging. It is estimated that BM occur in up to 30 percent of adult patients with solid malignancies (1). Consequently, the prevalence and incidence of BM are continuously rising, making BM a significant social and health problem. Until recently, due to limited therapeutic options, BM were typically treated in a standardized manner, with whole-brain radiotherapy (WBRT) being the primary treatment for decades. Current treatments for BM include surgery, stereotactic radiosurgery (SRS), WBRT, chemotherapy, and modern targeted therapy (2).

Although the role of WBRT in patients with brain metastases has evolved in recent years, and its usage has decreased, WBRT remains a crucial tool in the standard treatment for the majority of patients with multiple BM (3). While radiotherapy has made significant advancements in general, WBRT itself has not seen substantial changes in recent decades. It has long been recognized that WBRT can lead to serious, irreversible side effects on the central nervous system. Neurocognitive dysfunction has become an increasingly relevant concern in patients with BM who receive WBRT. Preserving a good quality of life (QoL) for as long as possible and minimizing potential iatrogenic side effects of treatment are currently top priorities, not only in palliative medicine (4).

Although cognitive impairment in patients with BM is likely influenced by multiple factors, post-radiation changes in the hippocampus are considered one of the primary factors affecting neurocognitive function (NCF), particularly memory, and ultimately overall QoL (5–7). This article presents multiple clinical and preclinical data on radiation-induced damage to neural progenitor cells located in the subgranular zone of the hippocampus and its impact on radiation-induced neurocognitive decline, specifically in terms of short-term memory formation and recall (8). Additionally, by systematic evaluation of relevant papers published on PubMed database over last 20 years, we provide a comprehensive overview of the various changes that can occur in the left or right hippocampus as a consequence of radiotherapy with consideration of the potential benefits of sparing unilateral hippocampus during WBRT in patients with multiple brain metastases (presented in section 4.2 and 4.3 after necessary gradual description of relevant implicatons).

## 2 Hippocampus – basic overview

Due to bilateral brain symmetry, the hippocampus is situated in each cerebral hemisphere. It can be simplified that if there is unilateral damage to the hippocampus, with the structure in the other hemisphere remaining intact, memory functions of the brain can generally remain almost normal (9). Conversely, severe damage to both hippocampi in both hemispheres can lead to significant difficulties in forming new memories. Nevertheless, clinical observations and numerous studies demonstrate that damage to different regions of the hippocampus can result in specific memory disorders. For instance, verbal memory retention is most likely associated with the anterior part of the left hippocampus, while the right hippocampus plays a more prominent role in executive functions and regulation during verbal memory retrieval. The posterior part of the left hippocampus could then be linked to verbal memory capacity (10). The findings of our previous research are consistent with these observations, as discussed in further details (11, 12).

The hippocampus does not appear to have a uniform structure along its longitudinal axis. There is evidence of differences in both structure and function. The anterior part of the hippocampus is preferentially connected to the amygdala and orbitofrontal cortex and is believed to be mainly involved in episodic memory and the mediation of anxiety-related behaviors. In contrast, the posterior part of the hippocampus is preferentially connected to the retrosplenial and posterior parietal cortex and is thought to be especially engaged in memory and spatial navigation (13). Other parts of the brain (amygdala, fornix, etc.) are involved in the neurocognitive function in the complex brain organization.

In addition to the functional distinctions between the anterior and posterior hippocampus, there is substantial evidence regarding the lateralization of hippocampal functioning, highlighting that the roles of the right and left hippocampus are not identical. This knowledge is derived from findings in patients with unilateral hippocampal lesions, including those with conditions such as schizophrenia and mild cognitive impairment, as well as individuals who have undergone unilateral hippocampal resection as a treatment for epilepsy. In broad terms, it can be asserted that spatial memory is primarily associated with the right hippocampus, while episodic memory is linked to the left hippocampus.

Furthermore, gender differences in hippocampal lateralization during spatial tasks have been observed, with greater activation on the left in females and on the right in males. This discrepancy is likely attributed to the fact that females tend to rely more on verbal strategies, whereas males exhibit a preference for nonverbal spatial strategies (14). Additionally, several other studies discussed below have described varying clinical significance in the postradiotherapy changes between the left and right hippocampus.

It has been shown that radiation-induced microstructural changes in the brain, which can subsequently lead to cognitive impairment, occur soon after radiation exposure. These alterations may occur without obvious radiographic manifestations and may be detectable, for example, by diffusion tensor imaging (DTI) as white matter changes. Further information about radiation damages can be provided by MR perfusion a diffusion-weighted imaging (DWI). MR spectroscopy (MRS) is able to evaluate radiation-induced brain injury by assessing the metabolic concentrations at the molecular levels (15, 16).

## 3 Laterality in hippocampal function

Much of the information on this topic has been gleaned from non-oncology patients or healthy volunteers. Patients with BM possess unique characteristics; their cognitive and neurological functions may be influenced by the oncology disease itself, typically resulting in a poor prognosis and short life expectancy. The primary treatment goal is to enhance or sustain the quality of life. The view of this issue from the point of view of radiation oncology must be somewhat different from, for example, epilepsy surgery. In neurobiology, it is understood that explicit memory is primarily housed in the brain’s temporal lobes, specifically within the hippocampus, as well as in the amygdala and neocortex.

While both episodic and spatial memory rely on the hippocampus, distinctions in these functions suggest partial separation and distinct structural neural foundations, as well as varying connections with other brain regions. Notably, the anterior and posterior hippocampus exhibit differences in structure and connectivity within the brain. Some studies suggest that the posterior hippocampus plays a greater role in spatial memory, whereas the anterior hippocampus is primarily associated with episodic memory (17, 18).

In addition to the functional differences between the anterior and posterior hippocampi, numerous examples support the presence of lateralization in hippocampal functions, meaning that the left and right hippocampus serve distinct functions. Much of this evidence is derived from studies involving patients with unilateral hippocampal lesions and unilateral hippocampal resections. Research on patients who have undergone resection of the left hippocampus for the treatment of epilepsy suggests impairment verbal memory tasks, specifically affecting learning and retention of story content, word recognition, recall, and verbal associative memory (19–21). In contrast, resections of the right hippocampus and parahippocampal cortex lead to deficits in visuospatial tasks (22). This aligns with findings of lateralized hippocampal activation in functional neuroimaging studies. Lateralization of hippocampal function is also evident in spatial memory, depending on whether verbalizable stimuli or abstract nonverbal stimuli are employed. This suggests possible differences in different cognitive strategies. These results provide support for the concept of functional lateralization within specific aspects of spatial memory (23).

**Figure 1:**
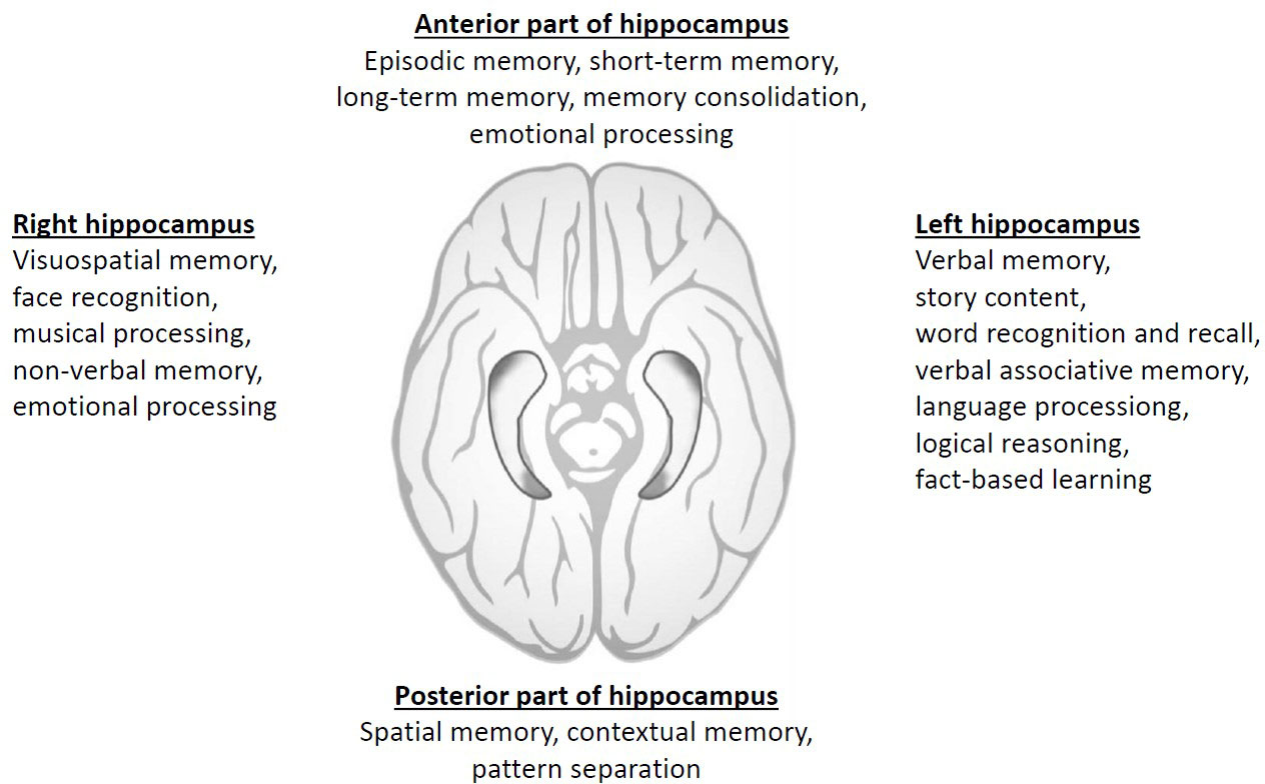
Summary of simplified division of different neurocognitive domains according to hippocampal location and laterality.

Consequently, it can be inferred that the previous hypothesis of strictly lateralized organization of brain functions, with verbal memory components residing exclusively in the left hemisphere and spatial memory components solely in the right hemisphere, may not be so pronounced (24). Thus, with the advancement of cognitive neuroscience, the idea of strict structural specificity is now being questioned (25).Thus, the specific neurocognitive functions attributed to the left and right hippocampus are not as clear-cut as a following **Figure 1** might suggest. While there are some general trends in terms of lateralization of functions in the brain, the hippocampus is a complex structure, and many functions involve both sides working together. Additionally, individual differences, as gender aspects, can play a significant role.

Conversely, the recovery and compensation of memory functions represent a demonstration of a particular functional plasticity within the brain (26). For instance, memory deficits typically associated with the contralateral temporal lobe function in patients with unilateral hippocampal sclerosis may show improvement after surgery (27). However, in patients with brain tumors, who are further burdened by oncological treatments, including radiation injury, it is not possible to assume the same ability for compensation.

From a radiotherapy perspective, the feasibility of sparing both hippocampi, only the right hippocampus, or only the left hippocampus is often influenced by the presence or proximity of individual BM within the hippocampus. The consideration of which functions are associated with each hippocampus is secondary in this context. Conversely, the discussed differences in lateralization are a significant factor to consider when contemplating unilateral hippocampal investigation in WBRT as discussed below. In this scenario, one of the hippocampi remains covered by full dose of radiation while only the other is protected. This experimental approach may offer greater sparing possibilities when focusing on a single hippocampus, along with improved rest of the brain irradiation (28).

### 3.1 Radiotherapy and hippocampus

The side effects of radiotherapy on the brain are highly specific to the tissues and structures involved. This specificity arises from the combined impact of radiation on brain vasculature, neuroglial cells, and their precursors, including stem cells. Radiation-induced inflammatory effects and the disruption of the blood-brain barrier also play a role (29).

The radiation-induced inflammatory response leads to an increased activation of microglia, which release cytokines like tumor necrosis factor-alpha and interleukin-1 beta. Radiation is particularly cytotoxic to proliferating neuroglial progenitor cells, disrupting both gliogenesis and neurogenesis and resulting in a reduction in the number of newly formed neurons. One region in the brain known for its neurogenic potential is the hippocampus, specifically the subgranular zone of the hippocampal gyrus dentatus, housing a niche of neural stem cells crucial for memory formation. In our previous work, we hypothesized that the loss of neuronal cells in the hippocampal region that occurs after irradiation can be measured by changes in N-acetylaspartate (NAA) concentration using MRS. Our results showed that after whole brain radiotherapy (WBRT), there was a decrease in NAA concentration in both hippocampi, and these changes were associated with a decline in cognitive function as assessed by a battery of neurocognitive tests focused on memory, including the Auditory Verbal Learning Test and the Short Test of Visual-Spatial Memory-Revised. We observed a moderate positive correlation between the decrease in NAA concentration in the left hippocampus and some subtests related to verbal memory (12). A radiation technique employing intensity-modulated RT (IMRT) to administer a therapeutic dose to the entire brain region while sparing the bilateral hippocampi is known as hippocampal avoidance whole brain radiotherapy (HA-WBRT) (30–33).

In a pivotal phase II trial (RTOG 0933), HA-WBRT in BM patients was linked to the preservation of tested cognitive function and reported quality of life compared to historical controls (31). Subsequently, the results of a randomized phase III study (NRG CC001, published in 2020) comparing HA-WBRT plus memantine, the N-methyl-D-aspartate (NMDA) receptor antagonist, to WBRT plus memantine in 518 BM patients demonstrated a significantly lower risk of cognitive failure (adjusted hazard ratio, 0.74; P = .02) with hippocampal sparing, while there was no difference in intracranial progression or overall survival. HA-WBRT in combination with memantine can now be considered a new standard of care for the treatment of multiple brain metastases (34).

However, the planning process for HA-WBRT is significantly more labor-intensive compared to traditional WBRT. The key challenge lies in accurately defining the target volume and identifying critical structures and organs at risk (OAR), such as the hippocampus and hippocampal-avoiding zones (33). To address this, a consensus-based atlas for contouring in Neuro-Oncology can help reduce inter- and intra-observer delineation variability (35). Recently, an MRI-based OAR autosegmentation atlases are developed as well. Autosegmentation allows for high-quality contouring in a limited time frame. The accuracy of hippocampal contouring in the HA-WBRT technique is enhanced through automatic hippocampal segmentation using multitasking learning (36).

Other areas that may be relatively sensitive to radiation include periventricular regions (such as the subventricular zone) and white matter tracts containing oligodendrocyte precursor cells. These areas are relevant to brain neuroplasticity, which pertains to the brain’s ability to establish or modify connections with other brain regions. Neuroplasticity is an essential property, particularly for brain injury recovery, among other functions.

The importance of preserving intact white matter integrity in maintaining cognitive function has also been highlighted in a secondary analysis of the RTOG 0933 trial. In their study, Bovi et al. established a correlation between neurocognitive decline and the pretreatment volume of MRI-determined white matter injury. They found a positive correlation (r = 0.54, P <.05) between a larger volume of pretreatment white matter injury and declines in recognition, as assessed by the Hopkins Verbal Learning Test-Revised (37).

A recently published prospective longitudinal trial assessed associations between changes in amygdala morphometry and functional outcomes in patients with primary brain tumors receiving radiation therapy. Radiation dependent atrophy in bilateral amygdalae was associated with poorer memory, mood, and emotional well-being.

Advanced radiotherapeutic techniques such as volumetric modulated arc therapy (VMAT) enable the simultaneous sparing of other limbic brain structures involved in cognitive function for patients undergoing WBRT. While hippocampal sparing is already common practice in many cancer centers, the feasibility of extending this approach has, thus far, only been tested at the planning study level. The process of preparing a radiation plan is more time-consuming, and the homogeneity of radiation with respect to PTV (Planning Target Volume) dose coverage may be lower. Implementing an extended sparing approach for certain brain regions carries the risk of potentially impacting oncologic outcomes, including intracranial control and subsequent overall survival. Therefore, prospective studies are deemed necessary (38).

On the other hand, as advancements in stereotactic radiotherapy delivery continue, one might argue that preserving various other parts of the brain is safe, even in cases with multiple brain metastases (e.g., more than 15 lesions), especially when regular brain MR imaging follow-ups are conducted (38). The left and right hippocampus, left and right amygdala, fornix, and corpus callosum are crucial neurocognitive structures, and it is reasonable to assume that sparing all of them is essential to maximize the preservation of neurocognitive function. Indeed, in the NRG CC001 study, approximately 50% of patients treated with HA-WBRT and memantine experienced neurocognitive decline (34). It is conceivable that sparing more than just the hippocampi is necessary, as is being explored in the concept of Memory Avoidance WBRT, currently under evaluation in an ongoing phase II clinical trial (39). The Memory Avoidance region encompasses the left and right hippocampus, left and right amygdala, fornix, and corpus callosum, with constraints set at D100% ≤ 9 Gy and D0.03 cm3 ≤ 16 Gy in standard prescription of 30Gy in 10 fractions. In a dosimetry study involving ten enrolled patients (none of whom had brain metastases within the memory sparing region), only two of them failed to meet the constraints for achieving near-maximal dose sparing, as priority was given to target coverage and homogeneity of target irradiation. Utilizing modern LINAC-based volumetric modulated arc therapy, it is indeed possible to create a homogeneous treatment plan while preserving all critical neurocognitive function-related structures (40). For the further development of this intriguing and innovative technique of Memory Sparing-WBRT, the evaluation of post-treatment neurocognitive function and the assessment of the risk of local failure will be crucial.

### 3.2 Laterality of hippocampal changes after RT

Designing appropriate strategies to reduce radiation dose to the hippocampus would be enhanced if suitable imaging methods could be discovered to detect hippocampal damage *in vivo* in patients with brain tumors after cranial irradiation. Magnetic resonance imaging is a widely utilized neuroimaging method and is also employed in cognitive neuroscience. It can be utilized to assess regional morphology and physiology, including pathological issues, in the entire brain or in its individual components.

An example would be hippocampal volume measured by structural MRI. The utility of this method has been clinically validated, as seen in conditions such as Alzheimer’s disease, temporal lobe epilepsy, and traumatic brain injury (40, 41). However, this technique has not yet been successfully employed as a biomarker for radiation-induced hippocampal volume loss (42). There is a notable correlation between the reduction in hippocampal volume and the administered radiation dose to the hippocampus. Nevertheless, at the lowest doses, the hippocampi appear to exhibit an adaptive increase in volume, suggesting a potential neuroplasticity effect. Consequently, it may be advisable to shield at least one hippocampus by administering the lowest feasible dose to preserve cognitive functions (43).

Recently, a systematic review was published, and a behavioral meta-analysis was conducted on the association between cognitive outcomes and multimodal MRI imaging in childhood medulloblastoma (MB) survivors. As summarized in the article, several studies have explored the link between hippocampal volume changes following radiotherapy and memory function (44). One study reported that smaller hippocampal volumes were associated with poorer verbal associative memory (45), while another study found a correlation between right hippocampal volume and learning, attention, and memory (42). In one of the included studies, significantly lower ADC (Apparent Diffusion Coefficient) levels were observed in the hippocampi of MB patients compared to the control group. This study highlights impaired hippocampal microstructure, which may lead to decreased memory performance in patients treated for MB.

The association between hippocampal volume and memory functions was also validated in the opposite direction, as demonstrated by a positive correlation between grey matter volume in the posterior hippocampus of London taxi drivers and their spatial memory, along with their navigational abilities (46).

Other studies show different changes occurring in the left and right hippocampus after irradiation as discussed below (**Table 1**).

**Table 1:** Summary of studies reporting different post-radiotherapy changes in left vs. right hippocampal region.

Author, year	Diagnosis	Number of patients	observation	Laterality of greater changes
Yang, 2022 (47)	Glioblastoma	133	Mean left hippocampus dose was significantly associated with post-radiotherapy decline in MMSE scores (p = 0.005), while the right hippocampus not.	L
van der Weide, 2022 (48)	Low grade gliomas	17	The subgroup with left-sided tumors performed significantly lower on verbal tests. In the subgroup with right-sided tumors, RT dose in the left cerebrum was related to lower verbal memory performance	L
Qiu, 2021 (49)	Nasopharyngeal carcinoma	146	RT-associated progressive radial diffusivity reduction in the left cingulate angular bundle correlated with progressive cognitive impairment post-RT	L
Haldbo-Classen, 2020 (50)	Primary brain tumors	78	High RT dose to the left hippocampus associated with impaired verbal learning and memory (p = 0.04). RT dose to the left hippocampus, left temporal lobe, left frontal lobe and total frontal lobe associated with verbal fluency impairment (p < 0.05) and doses to the thalamus and the left frontal lobe with impaired executive functioning	L
Goda, 2020 (51)	Benign or low-grade brain tumors	48	A mean dose of ≤30 Gy to the left hippocampus as a dose constraint for preserving intelligence quotient is suggested	L
Tringale, 2019 (52)	Primary brain tumors	27	Higher mean dose to the left temporal pole white matter was significantly associated with decreased fractional anisotropy.	L
Shi, 2018 (53)	Nasopharyngeal carcinoma	56	Compared to pre-treatment group, cortical volumes of gray matter were significantly smaller in the left hippocampus, the right pulvinar and the right middle temporal gyrus	L
Raghubar, 2018 (54)	Pediatric brain tumor	26	Word Pair delayed recall was significantly associated with whole brain and right hippocampus mean dose, Integral biological effective dose, and Generalized equivalent uniform dose; and left hippocampus Generalized equivalent uniform dose	L/R
Zureick, 2018 (55)	Pediatric brain tumor	70	A higher left hippocampal V20GyE (percentage of the volume of a particular anatomical region receiving at least a 20 gray equivalent) was correlated with a score decline in all 4 measures.	L
Kim, 2018 (56)	Primary brain tumors	26	The mean dose of the left hippocampus and bilateral hippocampi were significantly higher in patients showing deterioration of the Seoul Verbal Learning Test for total recall and Recognition than in those without deterioration.	L
Pospisil, 2017 (12)	Brain metastases	35	Moderate positive correlation was observed between left hippocampal N-acetyl aspartat concentration decrease and Auditory Verbal Learning Test_total recall decline as well as with delayed recall decline. No correlation between right hippocampus h-tNAA and memory decline (AVLT) was observed.	L
Simo, 2016 (57)	Brain metastases from small cell lung cancer	22	decrease in gray matter over time in the right subcortical regions, bilateral insular cortex, and superior temporal gyrus plus in the right parahippocampal gyrus and hippocampus	R
Bodensohn, 2016 (58)	High grade gliomas	44	In the ‘verbal memory test’ lower percentile ranks were achieved in left-sided tumors compared to right-sided tumors. a correlation was detected between decreased figural recognition and the radiation dose to the left hippocampus	L
Tsai, 2015 (59)	Brain metastases	40	dosimetric parameters specific to left sided hippocampus exerted an influence on immediate recall of verbal predicting patients’ neurocognitive decline after receiving HS-WBRT	L
Farjam, 2015 (60)	Low-grade glioma or benign tumor	27	vascular dose response in the left hippocampus of females correlated significantly with changes in memory function at 6 and 18-months post radiotherapy	L
Greenberger, 2014 (61)	Pediatric patients with low-grade gliomas	32	subgroup analysis indicated some significant decline in neurocognitive outcomes for young children (<7 years) and those with significant dose to the left temporal lobe/hippocampus	L
Peiffer, 2013 (62)	Primary brain tumor	57	Regions of adult neurogenesis primarily predicted cognition at %v40 (percent of region of interest receiving 40 Gy) except for the right hippocampus which predicted at %v10	R
Redmond, 2013 (63)	Pediatric primary brain tumors and controls	74	significant relationship between reduced performance on verbal learning and increasing dose to the cerebrum and reduced performance on visual perception and increasing dose to the left temporal lobe	L

**Figure 2:**
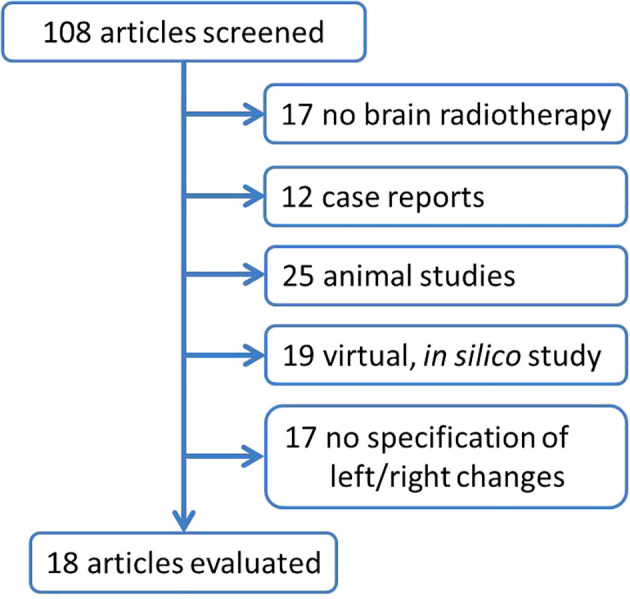
Flowchart summarizing the selection of studies describing different postradiotherapy changes in left vs. right hippocampal region. The search terms ,,hippocamp*”, ,,radioth*”, irrad*, left and right were used to search papers published during last 20 years (since 2003). In total, 108 articles was received using he search terms ,,(“hippocamp*”[All Fields]) AND ((radioth*) OR (irrad*)) AND ((left) OR (right))” with limitation for english written papers. Abstracts were reviewed and articles describing the other than brain cancer, case report, animal studies, virtual, in silico studies, studies with no specification of left vs. right changes were excluded.

PubMed database was used on 20 July 2023 to extract scientific articles describing different postradiotherapy changes in the leff, versus right hippocampus (**Figure 2**). Out of 108 identified articles, total of 18 studies was further evaluated (**Table 1**).

In patients whose left hippocampus received a mean dose of 30.7 Gy and 31 Gy, respectively, a statistically significant decrease in mean total performance quotient score of >10% was observed at 3 and 5 years after fractionated RT (benign, low-grade juvenile tumors), but no significant correlation was found with the doses received by the right hippocampus (51).

Higher doses to the left hippocampus can lead to significant impairment of verbal learning and memory; high doses to the left hippocampus and other structures on the left side of the brain (left temporal lobe, left frontal lobe, etc.) can result in impairment of verbal fluency, executive functions, and working memory speed as shown in a cross-sectional study of 78 primary brain tumor patients after radiotherapy (50).

In a prospective study, Zureick et al. explored the correlation between cognitive function and the dose received by the hippocampus in pediatric patients after proton irradiation. The results revealed a significant decrease in scores for delayed verbal memory and a borderline decrease for immediate verbal memory. However, no significant change was observed in scores for immediate and delayed visual memory. Furthermore, they identified a correlation between higher V20GyE (volume receiving 20 GyE or equivalent) on the left hippocampus and a decline in memory scores. Based on these findings, it is advisable to consider investigating the left hippocampus in pediatric brain tumor patients during proton/photon radiation therapy (55).

In other study, 40 cancer patients underwent HA-WBRT, and their neurocognitive functions were assessed before and four months after treatment. The results indicated stable hippocampus-dependent memory but significant associations between certain radiation doses to the hippocampus and verbal memory preservation. Specifically, lower radiation doses to the left hippocampus were linked to preserved immediate verbal memory (59).

In another study, eighty patients aged at least 6 years but less than 21 years with low-grade glioma were treated with RT to 54 Gy. On multivariate regression, after accounting for hydrocephalus, decline in short-delay recall was associated with the volume of right or left hippocampus receiving 40 Gy (V40 Gy) (64). This is an example of studies, where no difference between left and right postradiotherapy changes is presented.

### 3.3 Concept of unilateral hippocampal sparing

There is sufficient evidence supporting the crucial role of the hippocampus in both episodic and spatial memory functions. Numerous reports have documented that bilateral damage to this structure leads to severe memory impairments, often resulting in severe amnesia (9). Notably, the randomized NRG-CC001 trial demonstrated a reduced incidence of memory impairment when both hippocampi were spared during WBRT.

Whether the hippocampus sustains damage or protection during radiotherapy, such changes typically affect both hippocampi. Maintaining the integrity of both hippocampi is considered essential for normal cognitive function. However, the precise involvement of the dominant and non-dominant hemispheres’ hippocampi in specific neurocognitive functions remains incompletely understood. From a radiobiological perspective, the hippocampus cannot be viewed as a solely serial or parallel organ. In cases where there is evidence of metastatic involvement in one hippocampus, it is advisable to consider at least a unilateral or partial sparing of the hippocampal region possibly even just the amygdala region (65–67). This approach represents a compromise, aiming to preserve neurocognitive functions partially while achieving more uniform irradiation of the brain region. Such an approach could significantly expand the indications for hippocampal avoidance whole-brain radiation therapy (HA WBRT), even for patients with unilateral hippocampal metastasis involvement or metastases in close proximity to the hippocampi.

In cases where patients have multiple brain metastases, particularly when these are unfortunately situated within memory-related structures (as previously discussed regarding Memory Sparing WBRT), it may be advisable to consider sparing at least one hippocampal region. To be more specific, given the higher frequency of post-radiotherapy changes in the left hippocampus, a strategy involving the sparing of the dominant left hippocampus during WBRT could be considered as an alternative approach in the palliative radiotherapy of multiple BM.

### 3.4 Clinical implications and future directions

In our previous in-silico virtual planning study involving 10 patients, we developed radiation therapy treatment plans that incorporated unilateral left hippocampal sparing. Our aim was twofold: first, to maintain the same dosimetry for the left hippocampus as typically achieved in both hippocampal-avoiding WBRT to demonstrate improvements in brain target coverage, and second, to achieve the same left hippocampal dosimetry as usual but with only unilateral left hippocampal sparing to illustrate the potential for further reducing radiation dose to the spared left hippocampus (28).

With the implementation of unilateral left hippocampal sparing, we were able to achieve a significant reduction in brain radiotherapy homogeneity index. This approach also led to a decrease in near-maximal dose (D2%) to the brain and an increase in the near-minimal dose (D98%), thereby improving overall brain radiation dosage. Alternatively, by maintaining similar brain coverage, we could significantly reduce the radiation doses deposited in the left (spared) hippocampus (28).

**Figure 3:**
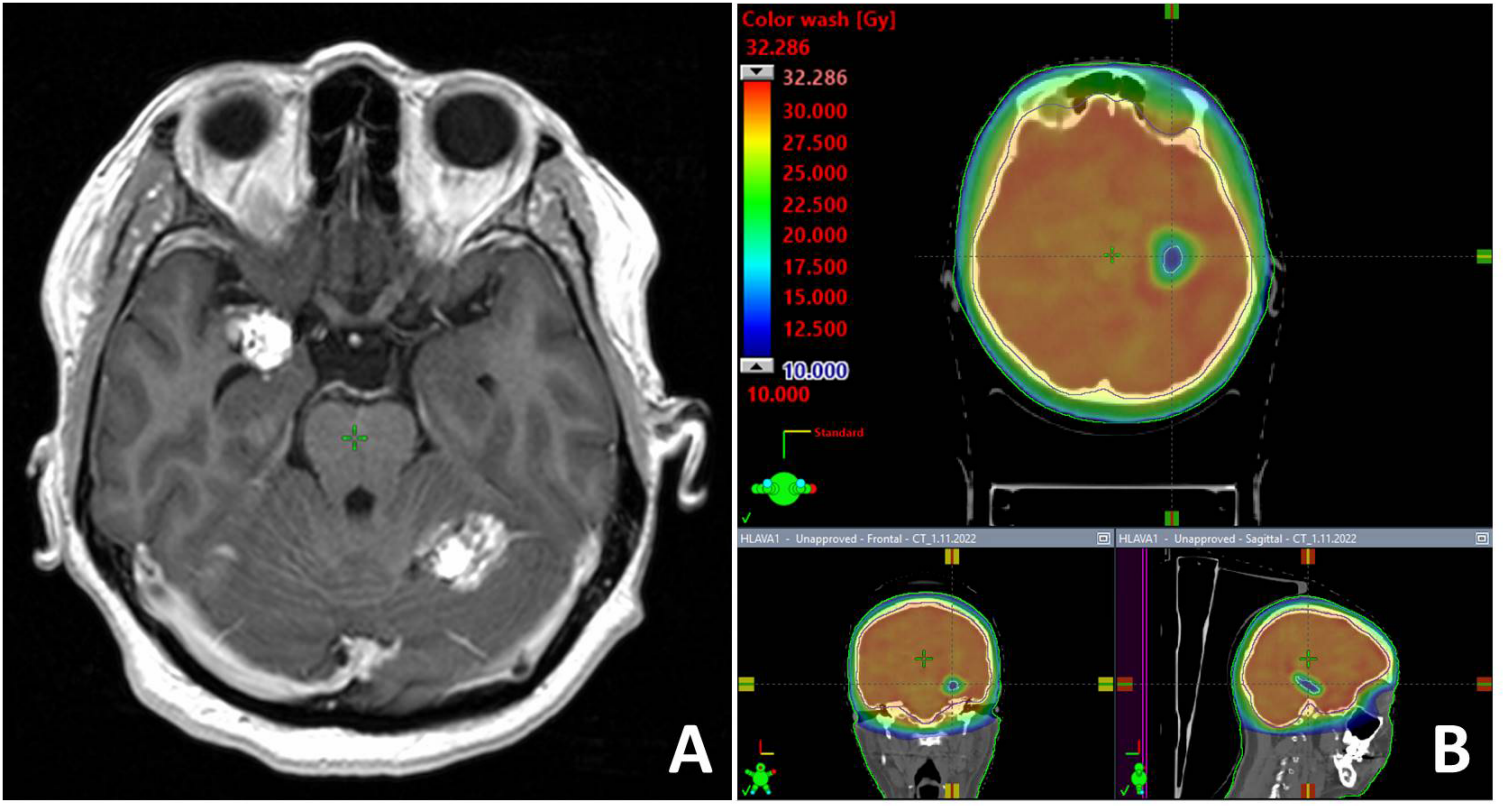
T1-weighted contrast enhancing MR examination with one metastasis located close to the right hippocampus **(A)**. Unilateral hippocampal sparing radiotherapy technique **(B)** was employed in the palliative RT.

**Figure 3** illustrates the case of 47-year old women with melanoma, who developed multifocal brain metastases presented supra- and infratentorially. One metastasis was presented in the close proximity to the right hippocampus. Unilateral left hippocampal sparing WBRT was performed with dose prescription to planning target volume 30Gy in 10 fractions. Dose within left hippocampus was reduced to D0.03Gy = 18.64Gy and to D100% = 9.65Gy.

The concept of partial hippocampal sparing in whole-brain radiation therapy (WBRT) has also been proposed by McKay et al. (66) and Sapienza et al. (65). Their work supports the idea of unilateral hippocampal sparing as a compromise approach. Additionally, their research suggests the potential for expanding the indications of hippocampal avoidance WBRT to include patients with unilateral metastatic involvement in the hippocampus.

The only currently ongoing and recruiting trial that focuses on unilateral hippocampal sparing during radiotherapy is NCT04801342, titled “Neurocognitive Outcome of Bilateral or Unilateral Hippocampal Avoidance WBRT With Memantine for Brain Metastases”. This phase 2 trial, conducted by researchers from National Taiwan University Hospital, involves enrolling patients with brain metastases located outside a 5-mm margin around either hippocampus (68). Patients are then randomized into two groups: the experimental arm, which receives unilateral hippocampal sparing WBRT plus memantine, and the active comparator arm, which undergoes bilateral hippocampal sparing WBRT plus memantine. In both cases, the prescribed dose is 10 fractions of 3.0 Gy each. The primary outcome of this study is the assessment of the decline in the Hopkins Verbal Learning Test-Revised (HVLT-R) memory score, which includes the sum of total recall and recognition index, measured from baseline to 6 months after the initiation of radiotherapy.

## 4 Conclusion

In summary, the human hippocampus plays a critical role in both intact episodic memory and spatial memory. Previous research indicates that there are notable structural and functional distinctions between the anterior and posterior regions of the hippocampus, reflecting differences in their connectivity to other brain regions. The posterior hippocampus is closely connected to the posterior parahippocampal cortex, which is involved in spatial function. This connectivity suggests that the posterior hippocampus is primarily responsible for spatial memory.

Conversely, the anterior hippocampus is associated with the perirhinal cortex, anterior temporal cortex, and amygdala, implying its involvement in episodic memory processes. It is worth noting that the activation pattern for episodic memory tasks is somewhat less distinct and tends to be more distributed in the left anterior hippocampus. However, the lateralization of activation may depend on the extent to which the task allows for the use of verbal strategies.

In essence, spatial tasks predominantly engage the right posterior hippocampus, while the engagement of the left anterior hippocampus is more prominent in episodic memory tasks, although this can vary based on the specific demands of the task, particularly in terms of verbal processing.

Differences in functional distribution along the longitudinal axis of the hippocampus, as well as lateral differences, could potentially account for sex differences in memory function. These distinctions might then manifest as variations in behavior between genders. However, the underlying neural mechanisms responsible for these sex differences remain largely unexplored. Gaining insight into the neural basis of sex differences in memory functions would not only contribute to our theoretical understanding of hippocampal function but also hold potential clinical significance. Notably, gender disparities in spatial memory performance are evident in variations in hippocampal activation patterns. Functional MRI studies have revealed greater right-sided activation in the posterior hippocampus among males. Furthermore, gender differences in the impact of unilateral hippocampal resection as a treatment for epilepsy have been observed, indicating that men and women may respond differently in terms of memory effects to this procedure.

Many patients with multiple BM are not suitable candidates for stereotactic radiotherapy, often due to the limited availability of advanced radiotherapy facilities and systems. Given that post-radiotherapy changes in the left hippocampus are more frequently associated with post-radiotherapy neurocognitive decline, the concept of unilateral left (dominant) hippocampal sparing has been proposed.

In addition to ongoing prospective clinical phase II trial (NCT04801342), it will be essential to routinely document specific doses administered to the left and right hippocampus. This documentation will help in comparing pre- and post-radiotherapy neurocognitive function. Determining the dominant hemisphere is crutial as well. Meanwhile, on an individual patient basis, unilateral (left, dominant) hippocampal sparing could expand the range of modifications available for whole-brain radiation therapy in multiple brain metastases unamenable for stereotactic radiotherapy.

## Author contributions

TK: Conceptualization, Funding acquisition, Supervision, Writing – original draft, Writing – review & editing. PP: Writing – original draft, Writing – review & editing. LHy: Data curation, Investigation, Writing – review & editing. LHn: Data curation, Investigation, Writing – review & editing. JM: Data curation, Investigation, Writing – review & editing. PS: Conceptualization, Supervision, Writing – review & editing.
